# Effects of intragastric administration of L-tryptophan on the glycaemic response to a nutrient drink in men with type 2 diabetes — impacts on gastric emptying, glucoregulatory hormones and glucose absorption

**DOI:** 10.1038/s41387-020-00146-9

**Published:** 2021-01-05

**Authors:** Maryam Hajishafiee, Rachel A. Elovaris, Karen L. Jones, Leonie K. Heilbronn, Michael Horowitz, Sally D. Poppitt, Christine Feinle-Bisset

**Affiliations:** 1 Adelaide Medical School and Centre of Research Excellence in Translating Nutritional Science to Good Health, University of Adelaide, Adelaide, Australia; 2 Lifelong Health Theme, South Australian Health and Medical Research Institute, Adelaide, Australia; 3 Endocrine and Metabolic Unit, Royal Adelaide Hospital, Adelaide, Australia; 4 Human Nutrition Unit, School of Biological Sciences, Department of Medicine, University of Auckland, Auckland, New Zealand

**Keywords:** Type 2 diabetes, Type 2 diabetes

## Abstract

**Background:**

The rate of gastric emptying and glucoregulatory hormones are key determinants of postprandial glycaemia. Intragastric administration of L-tryptophan slows gastric emptying and reduces the glycaemic response to a nutrient drink in lean individuals and those with obesity. We investigated whether tryptophan decreases postprandial glycaemia and slows gastric emptying in type 2 diabetes (T2D).

**Methods:**

Twelve men with T2D (age: 63 ± 2 years, HbA1c: 49.7 ± 2.5 mmol/mol, BMI: 30 ± 1 kg/m^2^) received, on three separate occasions, 3 g (‘Trp-3’) or 1.5 g (‘Trp-1.5’) tryptophan, or control (0.9% saline), intragastrically, in randomised, double-blind fashion, 30 min before a mixed-nutrient drink (500 kcal, 74 g carbohydrates), containing 3 g 3-O-methyl-D-glucose (3-OMG) to assess glucose absorption. Venous blood samples were obtained at baseline, after tryptophan, and for 2 h post-drink for measurements of plasma glucose, C-peptide, glucagon and 3-OMG. Gastric emptying of the drink was quantified using two-dimensional ultrasound.

**Results:**

Tryptophan alone stimulated C-peptide (*P* = 0.002) and glucagon (*P* = 0.04), but did not affect fasting glucose. In response to the drink, Trp-3 lowered plasma glucose from *t* = 15–30 min and from *t* = 30–45 min compared with control and Trp-1.5, respectively (both *P* < 0.05), with no differences in peak glucose between treatments. Gastric emptying tended to be slower after Trp-3, but not Trp-1.5, than control (*P* = 0.06). Plasma C-peptide, glucagon and 3-OMG increased on all days, with no major differences between treatments.

**Conclusions:**

In people with T2D, intragastric administration of 3 g tryptophan modestly slows gastric emptying, associated with a delayed rise, but not an overall lowering of, postprandial glucose.

## Introduction

Gastric emptying and the release of the glucoregulatory hormones, insulin and glucagon, are important determinants of the postprandial blood glucose response to carbohydrate-containing meals, in health, obesity and type 2 diabetes (T2D)^1^. For example, gastric emptying, which exhibits a substantial inter-individual variation, accounts for about 35% of the variance in peak postprandial blood glucose^1^. Slowing of gastric emptying, by dietary or pharmacological means, attenuates postprandial glycaemic excursions, particularly during the first 30–60 min postprandially^2,3^. Gastric emptying and the release of glucoregulatory hormones can be influenced by nutrients^4,5^. For example, in T2D, whey protein, when given as a preload 30 min before a high-carbohydrate meal, slowed gastric emptying and stimulated insulin, leading to a reduction in the glycaemic response^6^. These effects of protein appear to be mediated, at least in part, by their digestion products, amino acids — both circulating amino acids and as a result of the interaction of amino acids with the small intestine^7–10^.

A number of amino acids have been shown to reduce the blood glucose response to glucose or mixed-nutrient drinks^11–15^. The aromatic amino acid, tryptophan, is of particular interest; it potently stimulates pyloric motility and plasma cholecystokinin (CCK) concentrations, which are both central to the slowing of gastric emptying^16,17^. Solutions containing tryptophan, when administered intragastrically or orally, empty from the stomach more slowly than control solutions^18,19^. We reported that intragastric administration of 3 g tryptophan slowed gastric emptying of a mixed-nutrient drink, consumed 15 min later, associated with a reduced glycaemic response in the first 30 min, in both lean individuals and those with obesity who do not have T2D^15^. That intragastric tryptophan had a minimal^16^, or no^19^, effect to stimulate insulin, and may reduce insulin^15^, suggests that the attenuation in postprandial blood glucose is unlikely to reflect an insulinotropic effect of tryptophan. Like other amino acids, tryptophan has been reported to stimulate glucagon^15,16^, which may potentially counteract glucose lowering by stimulating glycogenolysis and gluconeogenesis. Hence, slowing of gastric emptying is likely to be fundamental to a reduction in postprandial glycaemia by tryptophan. The above considerations are of relevance to the management of T2D; it is now appreciated that postprandial glycaemic excursions are a major determinant of overall glycaemic control, as assessed by measurement of glycated haemoglobin, and the consequent risk of microvascular complications (i.e. retinopathy, nephropathy and neuropathy). However, there is no information about the effects of tryptophan in T2D. The observed reduction in postprandial glucose resulting from slowing of gastric emptying, induced by tryptophan, should intuitively be associated with a reduction in the rate of carbohydrate absorption, as supported by animal studies^20^. Glucose absorption can be measured in humans using plasma concentrations of the non-metabolisable glucose analogue, 3-orthomethyl-glucose (3-OMG)^21^.

The aim of the current study was to evaluate the effects of intragastric administration (to avoid potential confounding effects of taste) of tryptophan on the glycaemic response to, as well as glucoregulatory hormones and glucose absorption, and gastric emptying of, a mixed-nutrient drink in people with T2D.

## Materials and methods

### Participants

Twelve men with T2D (mean age: 63 ± 2 years, BMI: 30 ± 1 kg/m^2^, HbA1c: 49.7 ± 2.5 mmol/mol (6.7 ± 0.1%), duration of diabetes: 10 ± 2 years) participated in the study. Their diabetes was managed by metformin (500–2000 mg/day) alone (*n* = 7), or in combination with a DPP-4 inhibitor (*n* = 2) or a sodium-glucose transport inhibitor (*n* = 3). Participants were recruited by flyers placed at the Royal Adelaide Hospital and Diabetes South Australia, and through advertisements on online sites (University of Adelaide and Gumtree). Participants were excluded if they had significant gastrointestinal disorders or symptoms, cardiovascular or respiratory diseases or surgery, used medication known to affect gastrointestinal function and/or appetite, were lactose-intolerant, consumed protein supplements or >2 standard drinks (20 g) alcohol on >5 days a week, were vegetarians or smokers, or had an estimated glomerular filtration rate <45 mL/min, or low serum ferritin (<30 μg/L) or iron (<8 μmol/L) levels (a requirement by the Human Research Ethics Committee). In all cases, autonomic nerve function was evaluated using standardised cardiovascular reflex tests, and the presence of autonomic neuropathy (i.e. a score ≥3) represented an exclusion^22^. After inclusion, each participant was allocated to a treatment order of balanced randomisation (www.randomization.com) by a research officer who was not involved in data analysis. The Human Research Ethics Committee of the Central Adelaide Local Health Network approved the study protocol, and the study was performed in accordance with the Declaration of Helsinki and the National Health and Medical Research Council of Australia Statement on Ethical Conduct in Human Research. All participants provided written informed consent before inclusion. The study was registered as a clinical trial with the Australia and New Zealand Clinical Trials Registry (www.anzctr.org.au) (trial number: ACTRN12613000899741).

### Study design

We investigated the effects of intragastric administration of (i) 3 g (Trp-3) or (ii) 1.5 g (Trp-1.5) tryptophan, or (iii) control (0.9% saline) on plasma glucose (the primary outcome), C-peptide (a measure of insulin secretion), and glucagon concentrations and glucose absorption in response to, and gastric emptying of, a mixed-nutrient drink, consumed 30 min after the intragastric treatments.

### Control and tryptophan treatments

Tryptophan treatments were prepared by dissolving 1.5 g or 3 g food-grade tryptophan (PureBulk Inc., Roseburg, Oregon, USA), 58 mg CaCl_2_xH_2_O, and 1.75 g or 1.65 g NaCl, respectively, in 200-mL water for irrigation. Control solutions consisted of 58 mg CaCl_2_xH_2_O and 1.85 g NaCl in 200-mL water. The solutions were approximately iso-osmolar (control: 296, 1.5 g: 318, 3 g: 335 mOsm/kg) and had a pH of ~7 and a temperature of ~23 °C. Solutions were prepared by a research officer, who was not involved in the performance of the studies or data analysis, on the morning of each study and administered via a nasogastric catheter directly into the stomach. Syringes were covered, so that both study participants and the investigators performing the study were blinded to the nature of the solution. The doses of tryptophan were chosen based on our previous work and were well tolerated^15^.

### Study protocol

Participants were studied in a randomised, double-blind, cross-over fashion on three occasions each separated by 3–7 days. Participants were provided with a standardised meal (Beef lasagne, McCain Food, Wendouree, Victoria, Australia; energy content: 603 kcal), to be consumed between 6.30 p.m. and 7 p.m. on the night prior to each study. All participants were instructed to maintain their normal eating habits between study days and to discontinue their glucose-lowering medication for 48 h, and refrain from strenuous exercise and alcohol for 24 h, before each study. Participants were also asked to abstain from any solids or liquids, except water (which was allowed until 7 a.m.), after the evening meal until they attended the Clinical Research Facility at the University of Adelaide at 8.30 a.m. the next morning. An intravenous cannula was placed into a forearm vein, and the arm was kept warm with a heat pad for regular sampling of ‘arterialised’ blood. A baseline blood sample for measurement of plasma glucose and hormones, and a baseline antral area measurement, using two-dimensional (2D) ultrasound, with the participant seated in an upright position, were taken. Participants also completed a visual analogue scale (VAS) questionnaire to assess gastrointestinal symptoms. Each participant was then intubated with a soft-silicon feeding tube (outer diameter: 4 mm; Dentsleeve, Mississauga, Ontario, Canada), which was inserted through an anaesthetised nostril into the stomach. Immediately thereafter (*t* = −31 min, ~8.45 a.m.), participants received the 200-mL intragastric bolus of one of the two doses of tryptophan, or control, within 1 min. The tube was then removed. Further blood samples and VAS ratings were collected at *t* = −20, −10 and −1 min. At *t* = −1 min, each participant consumed, within 1 min, a mixed-nutrient drink (350 mL, 500 kcal, 74 g carbohydrates, including maltodextrin and sucrose, 18 g protein and 15 g fat) consisting of 325-mL Nestle Resource Plus^®^ vanilla (Nestle Healthcare Nutrition, Tongala, Victoria, Australia) plus 25-mL water to make up the final volume, containing 3 g 3-OMG (Sigma-Aldrich, Milwaukee, Wisconsin, USA). Blood samples and VAS ratings were collected subsequently at 15-min intervals from *t* = 15 to 60 min and at 30-min intervals from *t* = 60 to 120 min. Measurements of antral area were obtained immediately after the drink (*t* = 0 min), at 5-min intervals from *t* = 0 to 60 min, and at 15-min intervals from *t* = 60 to 120 min. At *t* = 120 min, the cannula was removed, and participants were provided with a light lunch, after which they were free to leave the laboratory.

### Measurements

#### Plasma glucose, C-peptide, glucagon and serum 3-OMG concentrations

For glucose and hormones, venous blood samples (7 mL) were collected into ice-chilled ethylenediaminetetraacetic acid-containing tubes. For serum 3-OMG, 3-mL venous samples were collected into untreated tubes and allowed to clot. Plasma and serum were each separated by centrifugation at 3200 rpm for 15 min at 4 °C within 15 min of collection and stored at −80 °C until subsequent analysis.

Plasma glucose concentrations (mmol/L) were quantified by the glucose oxidase method using a glucose analyser (YSI 2300 Stat Plus, Yellow Springs Instruments, Yellow Springs, Ohio, USA). Intra- and inter-assay coefficient variations (CVs) were ≤2%.

Plasma C-peptide concentrations (pmol/L) were measured by ELISA immunoassay (10-1136-01, Mercodia, Uppsala, Sweden). The minimum detectable limit was 15 pmol/L and the inter- and intra-assay CVs were 8.3% and 2.9%, respectively. C-peptide reflects endogenous insulin secretion, since it is not extracted by the liver and its half-life is longer than that of insulin^23^.

Plasma glucagon concentrations (pg/mL) were measured by radioimmunoassay (GL-32K, Millipore, Billerica, Massachusetts, USA). The minimum detectable limit was 15 pg/mL, and inter- and intra-assay CVs were 6.9% and 4.2%, respectively.

Serum 3-OMG concentrations (mmol/L) were measured by liquid chromatography and mass spectrometry, with a sensitivity of 0.0103 mmol/L^24^.

#### Gastric emptying

Gastric emptying was evaluated by measuring antral area using 2D ultrasound (Aloka SSD-650 CL; ALOKA Co. Ltd., Tokyo, Japan). Imaging was performed with the transducer positioned vertically to obtain a sagittal image of the antrum, with the superior mesenteric vein and the abdominal aorta in a longitudinal section^25^. Images were taken at the end of inspiration to minimise the effects of the motion of the stomach that occurs with regular breathing^26^. A region-of-interest was drawn around the cross-section of the antrum using in-built software. The intragastric retention of the meal, expressed as a percentage of the meal at *t* = 0 min, at any given time^25^, and the gastric half-emptying time (T50), defined as the time taken for 50% of the meal to empty from the stomach^27^, were calculated.

#### GI symptoms

Nausea and bloating were quantified using a VAS questionnaire^28^. The strength of each sensation was rated on a 100-mm horizontal line, where 0 mm represented ‘sensation not felt at all’ and 100 mm ‘sensation felt the greatest’. Participants were asked to indicate how they were feeling at each time point by placing a vertical mark on the 100-mm line.

### Data and statistical analysis

The number of participants included was determined by power calculations based on our previous studies^15,16^. We calculated that *n* = 12 participants would allow detection of a 1.0 mmol/L reduction in blood glucose at α = 0.05 with a power of 80%^15^.

Areas under the curve (AUCs) for plasma glucose, C-peptide, glucagon and VAS ratings were calculated using the trapezoidal rule, from *t* = −31 to −1 min to assess the effects of tryptophan alone, and from *t* = −1 to 120 min (*t* = 0 to 120 min for gastric emptying and serum 3-OMG) to assess the responses to the mixed-nutrient drink. AUCs were analysed using general linear model mixed-model analysis, with baseline values as a covariate. C-peptide and glucagon concentrations at *t* = −1 min (i.e. immediately pre-drink), which we reported previously to correlate with subsequent glycaemic control^15^, peak plasma glucose, time to peak glucose and gastric half-emptying time (T50) were analysed using repeated-measures analysis of variance (ANOVA). Post-hoc comparisons, adjusted for multiple comparisons by Bonferroni correction, using parametric tests, were performed when significant ANOVA effects were found. Statistical analysis was performed using SPSS software (version 25, IBM, Chicago, Illinois, USA). Differences were considered statistically significant at *P* ≤ 0.05, and 0.05 < *P* ≤ 0.1 as trend. All data are reported as mean ± SEM.

## Results

All participants completed the study and tolerated the study conditions well. There were no effects of treatment on nausea or bloating, neither in response to tryptophan alone nor after the mixed-nutrient drink (Table 1). No participant had evidence of autonomic nerve dysfunction (mean total score: 1.1 ± 0.1; range: 0–2).

**Table 1:** AUCs of plasma glucose, C-peptide and glucagon concentrations and VAS ratings in response to tryptophan alone (*t* = −31 to −1 min) and a mixed-nutrient drink (*t* = −1 to 120 min), and gastric emptying and serum 3-OMG responses to, the mixed-nutrient drink, ingested 30 min after intragastric administration of tryptophan, at doses of 3 g (Trp-3) or 1.5 g (Trp-1.5), or control.

	Treatments			*P* value (ANOVA)
	Control	Trp-1.5	Trp-3	
Plasma glucose				
AUC_−31 to −1 min_ (mmol/L ✕ min)	234 ± 1	233 ± 2	231 ± 1	NS
AUC_−1 to 120 min_ (mmol/L ✕ min)	1390 ± 52	1412 ± 44	1341 ± 42	NS
Peak (mmol/L)	13 ± 1	13 ± 1	13 ± 1	NS
Time to peak (min)	81 ± 8	82 ± 5	90 ± 6	NS
Gastric emptying				
AUC_0 to 120 min_ (% retention ✕ min)	5172 ± 637	5650 ± 532	6026 ± 662*	0.04
T50 (min)	34 ± 8	43 ± 8	49 ± 7	0.05
Plasma C-peptide				
AUC_−31 to −1 min_ (pmol/L ✕ min)	26,140 ± 335	28,986 ± 427**	28,651 ± 839*	0.002
AUC_−1 to 120 min_ (pmol/L ✕ min)	244,611 ± 21,630	269,441 ± 24,255*	237,282 ± 25,079	0.01
Plasma glucagon				
AUC_−31 to −1 min_ (pg/mL ✕ min)	1259 ± 14	1386 ± 50	1378 ± 47	0.04
AUC_−1 to 120 min_ (pg/mL ✕ min)	7513 ± 366	7967 ± 400	8457 ± 328	NS
Serum 3-OMG				
AUC_0 to 120 min_ (mmol/L ✕ min)	22 ± 2	23 ± 2	21 ± 2	NS
Nausea				
AUC_−31 to −1 min_ (mm ✕ min)	191 ± 36	135 ± 16	186 ± 67	NS
AUC_−1 to 120 min_ (mm ✕ min)	836 ± 619	846 ± 640	996 ± 662	NS
Bloating				
AUC_−31 to −1 min_ (mm ✕ min)	156 ± 53	157 ± 57	182 ± 74	NS
AUC_−1 to 120 min_ (mm ✕ min)	1147 ± 604	1467 ± 737	1780 ± 675	NS

Data are mean ± SEM, *n* = 12. AUCs of plasma glucose, gastric emptying, plasma C-peptide, glucagon, serum 3-OMG and VAS ratings, and T50 were analysed using general linear model mixed-model analysis, including baseline as a covariate, with adjustments for multiple comparisons made using Bonferroni correction.

*AUC* area under the cure, *VAS* visual analogue scale, *T50* gastric half-emptying time, *3-OMG* 3-O-methyl-D-glucose, *NS* not significant.

* Trend for difference from control (*P* = 0.06).

** Significantly different from control (*P* = 0.002).

### Plasma glucose

#### Effect of tryptophan alone

There was no effect of treatment on fasting plasma glucose.

#### Effect following the mixed-nutrient drink

**Fig. 1:**
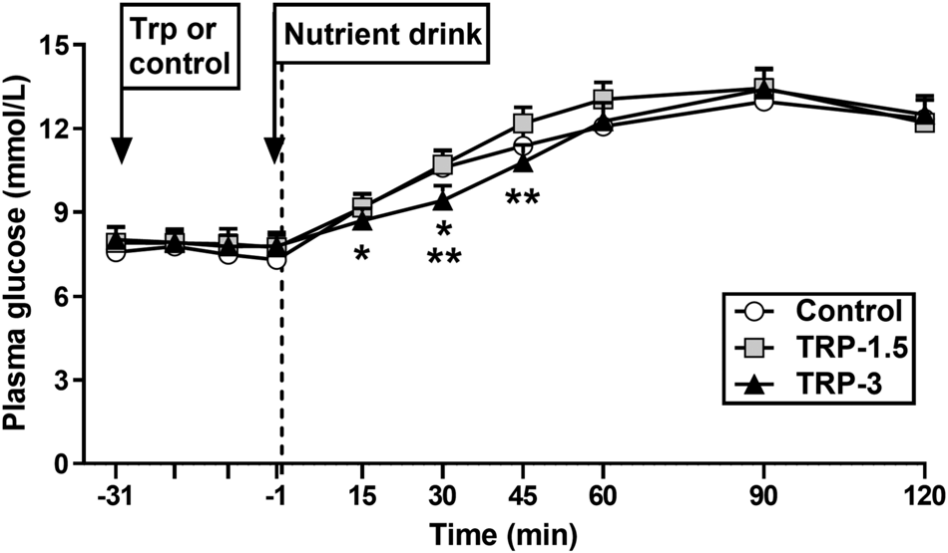
Plasma glucose concentrations following intragastric administration of 3 g (Trp-3) or 1.5 g (Trp-1.5) tryptophan, or control, in participants with type 2 diabetes. After tryptophan alone (i.e. *t* = −31 to −1 min), there was no effect on fasting plasma glucose. Following the mixed-nutrient drink (i.e. *t* = 0 to 120 min), there was no effect on overall plasma glucose AUC_−1 to 120 min_. However, there was a significant treatment * time interaction for plasma glucose (*P* = 0.05); Plasma glucose after Trp-3 was lower from *t* = 15 to 30 min compared with control (^*^P < 0.05), and from *t* = 30 to 45 min compared with Trp-1.5 (^**^P < 0.05). Data are expressed as mean ± SEM; *n* = 12. Data were analysed using general linear model mixed-model analysis, with baseline as covariate, and repeated-measures ANOVA, and adjustments for multiple comparisons were made using Bonferroni correction.

There was a substantial rise in plasma glucose after the drink on all days. While there was no effect of treatment on overall plasma glucose AUC_−1 to 120 min_, there was a treatment * time interaction for plasma glucose (*P* = 0.05). Plasma glucose was lower after Trp-3 from *t* = 15–30 min compared with control, and from *t* = 30–45 min compared with Trp-1.5 (*P* < 0.05 for all). There was no effect of treatment on peak glucose concentration or the time to peak concentration (Fig. 1 and Table 1).

### Gastric emptying

**Fig. 2:**
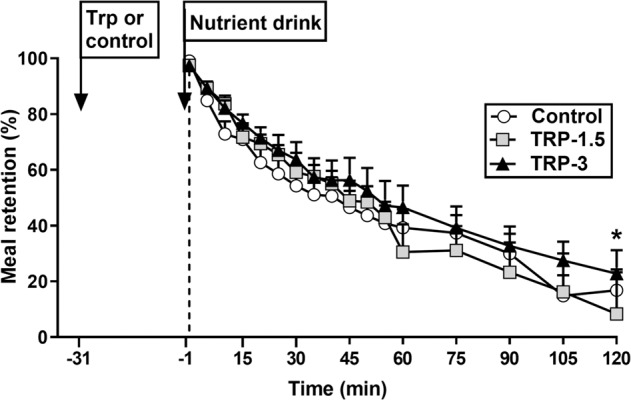
Gastric emptying, expressed as % drink retention, following intragastric administration of 3 g (Trp-3) or 1.5 g (Trp-1.5) tryptophan, or control, in participants with type 2 diabetes. There was an effect of treatment on gastric emptying AUC_0 to 120 min_ (*P* = 0.04), which tended to be greater after Trp-3 compared with control (^*^P = 0.06). There was also an effect of treatment on T50 (*P* = 0.05), although this was lost on post-hoc comparisons. Data are expressed as mean ± SEM; *n* = 12. Data were analysed using general linear model mixed-model analysis, with baseline as covariate, and adjustments for multiple comparisons made using Bonferroni correction.

There was an effect of treatment on gastric retention AUC_0 to 120 min_ (*P* = 0.04), which tended to be greater after Trp-3 compared with control (*P* = 0.06). While there was also an effect of treatment on T50 (*P* = 0.05), following post-hoc comparisons the difference between treatments was not significant (Fig. 2 and Table 1).

### Plasma C-peptide

#### Effect of tryptophan alone

There was an effect, albeit very small, of treatment on fasting C-peptide AUC_−31 to −1 min_ (*P* = 0.002), which tended to be greater after Trp-3 (*P* = 0.06), and was greater after Trp-1.5 (*P* = 0.002), compared with control. There was also an effect on C-peptide at *t* = −1 min (*P* = 0.01), which was greater after both Trp-3 and Trp-1.5 compared with control (*P* < 0.05 for both).

#### Effect following the mixed-nutrient drink

**Fig. 3:**
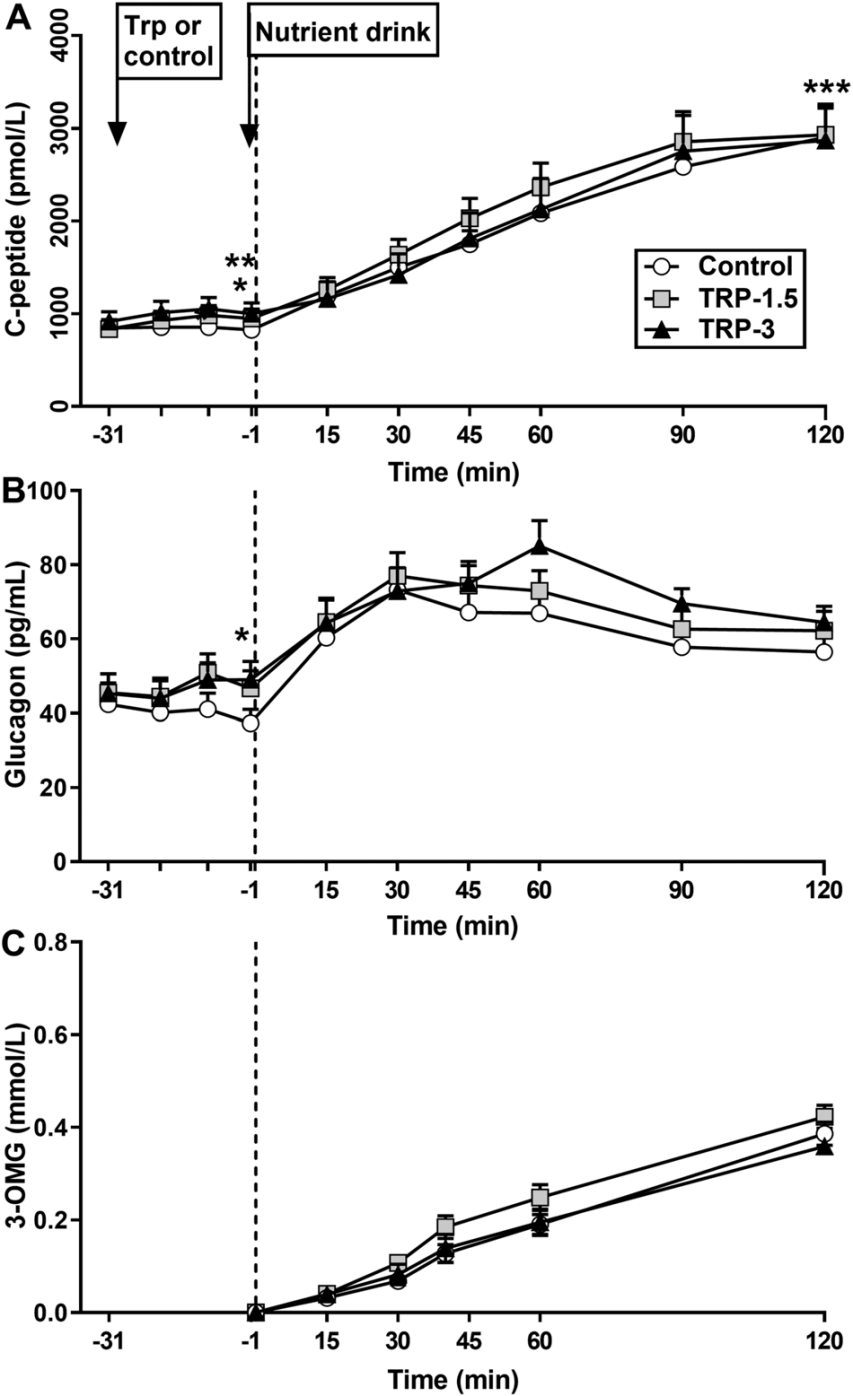
Plasma C-peptide, B glucagon and C serum 3-OMG concentrations following intragastric administration of 3 g (Trp-3) or 1.5 g (Trp-1.5) tryptophan, or control, in participants with type 2 diabetes. (**A**) After tryptophan alone (i.e. *t*=−31 to −1min), there was an effect of treatment on C-peptide AUC_−31 to −1min_ (*P*=0.002), which tended to be greater after Trp-3 (^*^P=0.06), and was greater after Trp-1.5 (^**^P=0.002), compared with control. There was also an effect on C-peptide at *t*=−1min (*P*=0.01), which was greater after both Trp-3 and Trp-1.5 compared with control (^*^P<0.05 for both). Following the mixed-nutrient drink (i.e. *t*=0 to 120min), there was an effect of treatment on C-peptide AUC_−1 to 120min_ (*P*=0.01), which tended to be greater after Trp-1.5, but not Trp-3 (^***^P=0.06). (**B**) After tryptophan alone, there was an effect of treatment on fasting glucagon AUC_−31 to −1min_ (*P*=0.04), however, this was lost on post-hoc comparisons. There was also an effect on glucagon at *t*=−1min (*P*=0.007), which tended to be greater after Trp-3 compared with control (^*^P=0.07). Following the mixed-nutrient drink, plasma glucagon rose on all days. However, there was no effect of treatment on glucagon AUC_−1 to 120min_, or a treatment*time interaction. (**C**) There was no effect of treatment on serum 3-OMG AUC_0 to 120min_, or a treatment*time interaction. Data are expressed as mean±SEM; *n*=12. Data were analysed using general linear model mixed-model analysis, with baseline as covariate, and repeated-measures ANOVA, adjusted for multiple comparisons using Bonferroni correction.

C-peptide rose substantially after the drink on all days. There was an effect of treatment on C-peptide AUC_−1 to 120 min_ (*P* = 0.01), which tended to be greater after Trp-1.5 (*P* = 0.06), but not Trp-3, compared with control. There was no treatment * time interaction (Fig. 3A and Table 1).

### Plasma glucagon

#### Effect of tryptophan alone

There was an effect of treatment on fasting glucagon AUC_−31 to −1 min_ (*P* = 0.04), however, this was lost on post-hoc comparisons. There was also an effect on glucagon at *t* = −1 min (*P* = 0.007), which tended to be greater after Trp-3 compared with control (*P* = 0.07).

#### Effect following the mixed-nutrient drink

There was a marked rise in plasma glucagon after the drink on all days. However, there was no effect of treatment on glucagon AUC_−1 to 120 min_, or a treatment * time interaction (Fig. 3B and Table 1).

### Serum 3-OMG

There was no effect of treatment on serum 3-OMG AUC_0 to 120 min_, or a treatment * time interaction (Fig. 3C and Table 1).

## Discussion

Our study indicates that in T2D, intragastric administration of tryptophan, at a dose of 3 g, but not 1.5 g, delays the rise in, but does not result in an overall lowering of, the plasma glucose response to a carbohydrate-containing mixed-nutrient drink, contrasting our previous observation in lean individuals or those with obesity^15^. Tryptophan slowed gastric emptying modestly, in line with our recent findings in lean people and normoglycaemic people with obesity^15^, which was likely responsible for delaying the rise in postprandial plasma glucose. Tryptophan also stimulated C-peptide, reflecting insulin secretion (although this effect was very small), as well as glucagon; the latter, therefore, most likely counteracted any glucose-lowering effect of insulin.

Protein ‘preloads’ containing whey^6^ and oral administration of specific amino acids^29^ have been shown to diminish postprandial glycaemic excursions in T2D and, accordingly, have the potential to be used in the management of T2D. We have reported that Trp-3, but not Trp-1.5, modestly lowered blood glucose in response to a mixed-nutrient drink in lean people and those with obesity^15^. We, therefore, hypothesised that, in people with T2D, in whom postprandial blood glucose is characteristically elevated, the blood glucose-lowering effect of tryptophan would be enhanced. Contrary to our hypothesis, tryptophan did not diminish the overall postprandial blood glucose, at either dose. However, Trp-3 modestly reduced the initial glycaemic response to the drink, by delaying the rise in blood glucose.

Gastric emptying is a major determinant of the glycaemic response to carbohydrate-containing meals in health, obesity and T2D^1^, and our previous study showed that Trp-3, administered intragastrically, slowed gastric emptying in both lean people and those with obesity^15^. Accordingly, the finding that tryptophan, in the same dose, slowed gastric emptying in people with T2D is consistent with these observations and is likely to account for the delayed rise in blood glucose. It needs to be recognised, though, that the magnitude of the slowing of gastric emptying was modest, which may explain the lack of effect on blood glucose lowering. The slowing of gastric emptying after Trp-3 may explain the apparent lack of effect of tryptophan on glucose absorption, as measured by serum 3-OMG. That is, any effect of Trp-3 on glucose absorption may have been masked by its effect to slow gastric emptying. Because our study did not include healthy participants as controls, the effect of tryptophan on glucose absorption in health, and whether any effect of tryptophan on glucose absorption may be altered in T2D, remains unclear.

Trp-3 tended to stimulate, and Trp-1.5 had a small effect to increase, plasma C-peptide immediately before the nutrient drink. We have reported that intraduodenal tryptophan, at a load of 0.15 kcal/min, providing ~3.3 g over 90 min, had a small effect to stimulate insulin in health^16^. This stimulation of insulin may reflect a direct effect of circulating amino acids^29^ or be secondary to the secretion of the incretin hormones, glucagon-like peptide-1 (GLP-1) or glucose-dependent insulinotropic polypeptide (GIP), which both have glucose-dependent insulinotropic effects^30,31^. The insulin-stimulatory effect of GIP is known to be markedly attenuated in T2D^32^. In healthy participants, the small insulinotropic effect of tryptophan is likely to be independent of GLP-1 and GIP^16^, as insulin stimulation occurred at blood glucose levels of <8 mmol/L^33^. After the drink, Trp-1.5, but not Trp-3, tended to increase C-peptide compared with control; this may reflect the lack of effect of Trp-1.5 to slow gastric emptying. Nevertheless, overall the insulinotropic capacity of tryptophan in the doses examined is modest. Therefore, the absence of overall glucose lowering by tryptophan is most likely due to only small insulin stimulation and modest slowing of gastric emptying, combined with relatively more potent stimulation of glucagon, possibly as a direct response to circulating amino acids^29^. A progressive rise in glucagon for ~30 min, followed by a sustained elevation, was evident on all three days, consistent with the well-established failure of postprandial glucagon suppression characteristic of T2D^34^.

The potential mechanism(s) underlying the differences in effects of tryptophan observed in this study, compared with those in healthy people^15^, deserve some consideration. Tryptophan may modulate GI motility, and, thus, gastric emptying, locally via its metabolite, serotonin^35^, or via CCK^16,36^. Tryptophan-induced stimulation of CCK most likely involves Ca^2+^-sensing receptors (CaSR) on enteroendocrine L cells^37^. Whether tryptophan-induced stimulation of CCK is altered in T2D warrants evaluation in future studies. The effect of tryptophan to stimulate insulin may be directly at the pancreatic beta-cell via activation of G protein-coupled receptor 142 (GPR142) and/or CaSR^38–40^, and may also involve serotonin^41^. Since people with T2D have beta-cell dysfunction, it is possible that the effects of tryptophan on one or more of these mechanisms may be attenuated, or the expression of these receptors altered, in T2D^42^. This warrants further investigation.

Some potential limitations of our study should be appreciated. We deliberately studied patients with well-controlled T2D (i.e. glycated haemoglobin is more dependent on postprandial glucose) without autonomic neuropathy and managed on oral hypoglycaemic drugs, but not insulin, as we considered that a glucose-lowering effect of tryptophan would be most evident in this group. It would be anticipated that in the cohort studied, the rate of gastric emptying would be normal, or slightly accelerated^1^. The two doses of tryptophan were based on our previous studies; while it is tempting to speculate that larger doses may have more potent effects, our previous studies have indicated that these may be associated with adverse effects^16^. Our study utilised intragastric administration of tryptophan, however, administration in capsules is also possible^43^, providing for a more practical application outside the laboratory setting. We only included men to avoid any effects of the menstrual cycle on gastric emptying^44^, thus, extrapolation of our observations to females should, of necessity, be circumspect.

In conclusion, our study suggests that in T2D, administration of tryptophan at a dose of 3 g before a carbohydrate-containing drink delays the rise in plasma glucose, probably as a result of slowing of gastric emptying, but does not affect the overall blood glucose response.
